# Investigation of natural infection of BALB C mice by *Bartonella henselae*

**DOI:** 10.1016/j.bjid.2024.104483

**Published:** 2024-11-29

**Authors:** Luciene Silva dos Santos, Sayros Akyro Soares Martins, Francine Ramos Scheffer, Alexandre Seiji Maekawa, Rafaela de Paula Silva, Gabriel Rabelo de Araújo, Paulo Eduardo Neves Ferreira Velho, Marina Rovani Drummond

**Affiliations:** a Universidade Estadual de Campinas (UNICAMP), Faculdade de Ciências Médicas (FCM), Laboratório de Pesquisa Aplicada Dermatologia e Infecção por Bartonella, Campinas, SP, Brazil; b Memorial University of Newfoundland, Faculty of Medicine – Endocrinology, St. John's, Newfoundland and Labrador, Canada; c Universidade Estadual de Campinas (UNICAMP), Departamento de Medicina, Divisão de Dermatologia, Campinas, SP, Brazil

**Keywords:** Mice, Inbred balb/c, Specific pathogen-free organisms, *Bartonella henselae*

## Abstract

Specific Pathogen-Free (SPF) animals are bred and maintained to exclude pathogens associated with significant morbidity or mortality, which may pose a risk to research replicability. The BALB/c strain is distributed globally and is among the most commonly used inbred strains in immunology and infectious disease research. Despite being a widely distributed bacterium that causes chronic infection, *Bartonella henselae* infection has not been investigated in any protocol that characterizes SPF animals. The objective of this study was to investigate the potential natural infection of laboratory animals of the BALB/c lineage by *B. henselae*. To achieve this, ten immunocompetent BALB/c mice were obtained directly from the bioterium and euthanized for collection of samples, including blood, skin, spleen, liver, heart, eye, kidney, intestine, esophagus, and brain. DNA was extracted using a commercial kit and tested via nested PCR for the *ftsZ* gene, as well as conventional PCR and qualitative real-time PCR using Sybr® Green for the citrate synthase gene (*gltA*), all specific reactions for *B. henselae*. All animals showed detection of *B. henselae* DNA in at least two different reactions in different tissues. The sequenced amplicons showed 100 % similarity to *B. henselae*. The use of mice infected by *B. henselae* in experiments is undesirable, as the bacteria can affect several aspects of the animal's physiology and consequently influence the results of the project, especially when subjected to immunosuppression. More studies are needed to understand and confirm the natural infection in experimental animals by *Bartonella* spp.. To date, no additional published reports of contamination of experimental animals by these bacteria have been identified.

## Introduction

Specific Pathogen-Free (SPF) animals are animals that are bred and managed to exclude pathogens associated with significant morbidity or mortality that may pose a risk research replicability. Generating and maintaining SPF animals requires detailed biosecurity planning for control of housing, environmental, and husbandry factors and a history of regimented pathogen testing.^1^, ^2^, ^3^

Criteria to claim SPF status for a lab animal species is not universally specified. Besides this, the Federation of European Laboratory Animal Science Associations (FELASA) developed a guideline for the accreditation of Health Monitoring programs. They emphasize that is important that animals are free of agents that may interfere with specific models or projects.^2^, ^3^, ^4^

However, there are several suppliers of SPF small laboratory mammals that make extensive health reports available, with test results for pathogens excluded from SPF stocks. Each vendor provides the pathogen status of their animals, level of barrier facility, along with testing procedures and frequencies.^3^^,^ ^5^

This is a list of the pathogens that should be excluded from SPF laboratory rodent colonies that includes bacteria, virus parasites, and fungi, as observed in Table 1 adapted from Murray et al., 2022.^3^^,^^5^^,^^6^

**Table 1:** Pathogens excluded from SPF laboratory rodent colonies.

Bacteria	Viruses	Parasites & fungi
*Helicobacter* spp.	Mouse hepatitis virus	*Encephalitozoon cuniculi*
*Pasteurella pneumotropica*	Mouse rotavirus	Ectoparasites (fleas, lice, mites)
Streptococci ßhaemolytic (not group D)	Murine norovirus	Endoparasites (tapeworms, pinworms, and other helminths)
	Parvoviruses (Minute virus of mice,	
Streptococcus pneumoniae	Mouse parvovirus, Kilham rat virus,	
*Citrobacter rodentium*	Rat minute virus, Rat parvovirus, Toolan's H-1 virus)	Protozoa (i.e., *Emeria* spp.),
*Clostridium piliforme*		
*Corynebacterium kutscheri*	Theiler's murine encephalomyelitis virus	*Toxoplasma gondii*
*Mycoplasma pulmonis*	Lymphocytic choriomeningitis virus	*Pneumocystis* spp.
*Salmonella* spp.	Mouse adenovirus type 1 (FL)	
*Streptobacillus moniliformis*	Mouse adenovirus type 2 (K87)	
Cilia-associated respiratory bacillus	Mousepox (ectromelia) virus	
*Klebsiella oxytoca*	Pneumonia virus of mice	
*Klebsiella pneumoniae*	Reovirus type 3	
*Pseudomonas aeruginosa*	Sendai virus	
*Staphylococcus aureus*	Hantaviruses	
*Bordetella bronchiseptica*	Herpesviruses (mouse cytomegalovirus,	
*Corynebacterium kutscher*	mouse thymic virus)	
	Lactatedehydrogenase elevating virus	
	Polyomaviruses (mouse polyomavirus, K virus)	
	Rat coronavirus/ Sialodacryoadenitis virus	
	Rat theilovirus	

Table adapted from Murray et al., 2022.

The authors could not identify any protocols that include investigation of *Bartonella henselae* infection to characterize SPF animals, despite the worldwide distribution of these bacteria, which cause chronic infection, including in mice.^7^

This species is the most important in human medicine and is also associated with infections in cats and dogs.^8^

Among the most widely used inbred SPF small laboratory mammals is the BALB/c mouse strain, which is distributed globally and is utilized in biomedical research, particularly in immunology and infectious disease research.^9^, ^10^, ^11^

Our objective was to investigate the possibility of natural infection of laboratory BALB/c mice by *B. henselae*.

## Methods

After authorization from the Animal Use Ethics Committee of the State University of Campinas (UNICAMP) under protocol number 4908-1/2018, 10 isogenic female BALB/cAnUnib mice (BALB/c), eight weeks old and weighing approximately 20 grams, were obtained directly from the Multidisciplinary Center for Biological Research (CEMIB) at UNICAMP. The name Unib was determined after consanguineous mating for 20 generations of the lineage originating from the Zentralinstitut für Versuchstierzucht (ZFV) – Hannover, Germany (BALB/cAn), according to the information from the Cemib website.^12^

Euthanasia was performed using Carbon Dioxide (CO_2_) for anesthesia followed by exsanguination via cardiac puncture.^13^ Tissue samples from blood, skin, spleen, liver, heart, eye, kidney, intestine, esophagus, and brain were collected from the mice. All sample collection was conducted in a laboratory that does not handle *Bartonella henselae* strains or *B. henselae* DNA, minimizing the risk of contamination. DNA extractions from the mice tissues were performed using the QIAamp DNA Mini Kit (Qiagen®), following the manufacturer's protocol.

**Fig. 1:**
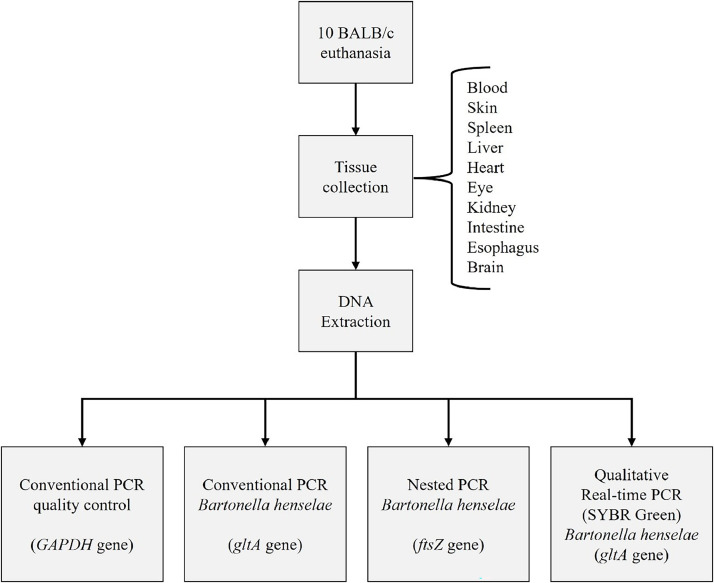
Flowchart of the performed procedures.

Molecular techniques were conducted at the Laboratory of Applied Research in Dermatology and Bartonella Infection at UNICAMP. We have three separate rooms to prevent DNA contamination. A uni-directional workflow is strictly enforced between pre-PCR areas (sample handling, PCR set up, DNA extraction) and post-PCR areas (DNA amplification, gel analysis, and amplicon purification). Dedicated sets of equipment, pipettes, and supplies were used in each of these locations. Strict laboratory procedures are implemented to prevent potential contamination of reagents and samples with amplicons. Different negative controls at different stages are included in every experiment to prevent PCR contamination.^14^ We followed the flow described in Fig. 1.

After DNA extraction using QIAamp DNA Mini Kit (Qiagen®), DNA was tested by conventional PCR for the endogenous gene targeting GAPDH (Glyceraldehyde-3-Phosphate Dehydrogenase gene). This procedure aims to assess the quality of the extracted DNA and verify the absence of amplification inhibitors.

Subsequently, the extracted DNA from the ten tissue samples was subjected to different PCR assays: nested PCR targeting the *ftsZ* gene (specific for *B. henselae*), qualitative SYBR®Green real-time PCR, and conventional PCR targeting the citrate synthase gene (*gltA*), as previously described. In real-time PCR, primers specific for the target gene encoding the citrate synthase (*gltA*) were used in the SYBR Green system, also specific for *B. henselae*. The results were interpreted qualitatively, considering them as either positive or negative.^14^

All PCR products were analyzed by electrophoresis on a 2 % agarose gel stained with GelRed® and visualized under ultraviolet light.

## Results

All samples tested positive for the endogenous gene by PCR, indicating the presence and integrity of the DNA, as well as the absence of inhibitors.

*B. henselae* DNA was detected in all ten mice of the BALB/c strain from CEMIB (Table 2).

**Table 2:** Results of *Bartonella henselae*-DNA detection in tissues of tested mice by species specific conventional, nested and real-time reactions.

Animal	Blood	Skin	Spleen	Liver	Kidney	Brain	Intestine	Esophagus	Eye	Heart	Number of positives tissues
Balb 1	+ CONV.	–	+ NESTED	+ NESTED	+ qlPCR	+ NESTED	–	–	+ qlPCR	–	6
									+ NESTED		
Balb 2	+ CONV.	–	–	–	+ NESTED	–	–	–	+ CONV.	–	3
Balb 3	–	–	–	+ NESTED	–	+ CONV.	+ CONV.	–	+ CONV.	–	4
							+ NESTED				
Balb 4	+ CONV.	–	+ NESTED	–	+ qlPCR	–	–	–	+ CONV.	–	4
	+ qlPCR								+ qlPCR		
									+ NESTED		
Balb 5	+ qlPCR	–	+ NESTED	–	+ CONV.	+ CONV.	–	–	–	–	4
	+ NESTED				+ qlPCR	+ qlPCR					
					+ NESTED	+ NESTED					
Balb 6	–	–	–	–	+ CONV.	–	–	–	+ qlPCR	–	2
									+ NESTED		
Balb 7	–	–	+ NESTED	+ NESTED	–	+ CONV.	+ NESTED	–	–	–	4
Balb 8	+ CONV.	–	–	–	+ qlPCR	–	–	–	–	–	2
	+ qlPCR										
Balb 9	+ qlPCR	–	–	–	+ CONV.	+ CONV.	+ NESTED	–	–	–	4
	+ NESTED				+ qlPCR						
					+ NESTED						
Balb 10	–	–	+ NESTED	–	–	+ qlPCR	–	–	–	–	2
						+ NESTED					

CONV = Conventional PCR for *Bartonella henselae* (*gltA* gene); NESTED = Nested PCR for *Bartonella henselae* (*ftsZ* gene); qlPCR = Qualitative Real Time qlPCR (SYBR Green) *Bartonella henselae* (*gltA* gene); – = negative; + = positive.

The amplicons of the second nested PCR reaction from spleen samples from animals 4, 7 and 10 were sequenced and showed 100 % similarity with *B. henselae* (Bartonella henselae strain Houston-I chromosome, complete genome CP020742.1).

## Discussion

We successfully detected *B. henselae* DNA in all ten SPF mouse samples from CEMIB, with each sample showing bacterium DNA detected by at least two different PCR assays across various tissues.

CEMIB serve as the central bioterium of the University and provides services to UNICAMP and External Institutions. The Center also provides Advisory Services to UNICAMP Units and Other National and Latin American Institutions. CEMIB was designed and built according to international standards for Reference Centers.^15^ The Center is part of the Laboratory Animal Quality Network of the International Council for Laboratory Animal Science. The building ensures that mouse and rat colonies are created within an efficient barrier system that prevents contamination. The Center has a Health Monitoring Program to evaluate the health status of mice and rat colonies through several diagnostic methods to detect viral, bacterial and parasitic infectious agents.^16^

The criteria for small laboratory mammals to claim SPF status is not specified or documented by entities that oversee lab animal health. The background infection can significantly impact treatment response, poising challenges for research replicability.^3^^,^^17^ *B. henselae*, although widely distributed, has not been systematically tested among pathogens from SPF animals.^3^ This limitation in pathogen screening protocols unfortunately contributes to the detection of bacterial infections such as this.

The Edouard et al. criteria for confirming *Bartonella* sp. infection diagnosis using PCR define that only samples with the DNA of the bacterium detected in at least two different genome regions should be considered positive.^18^

This approach reduces false positives and increases specificity. Even when applying these criteria, we detected *B. henselae* DNA in nine animals (only animal number 8 had not *fstZ* gene detection with nested PCR and the detection were with two different PCRs targeting *gltA* gene).

The presence of infected mice available for experiments poses challenges, as the bacterium can adversely affect various aspects of the contaminated organism, particularly under immunosuppression.^19^ This can significantly influence study results, especially in immunocompromised or genetically susceptible animals.

The natural infection of these animals may have occurred through the transplacental route from matrices obtained from foreign suppliers, as research on these bacteria in experimental animals remains limited.^7^^,^^20^

Vertical transmission of *Bartonella* spp. has been reported in small rodents, cats, horses, and humans.^7^^,^^21^, ^22^, ^23^, ^24^, ^25^ However, molecular tests for *Bartonella* spp. detection exhibit low sensitivity, often resulting in amplification in only one of the PCR assays. Only four of the 35 different positive samples amplified in all three PCRs performed. The low sensitivity of molecular reactions has been discussed in a recent study of our group.^14^

The authors were unable to find other studies on the contamination of experimental animals by *Bartonella* spp., highlighting the need for further research to understand and confirm natural infections in experimental animals by *Bartonella* spp. strains, including other lineages.

## Data availability

Data generated or analyzed during this study are available from the corresponding author upon reasonable request.

## Funding

Doctoral scholarship by The Brazilian National Council for Scientific and Technological Development (CNPq) 170501/2018-3 (Santos, LS); Research Assistant level III by the São Paulo Research Foundation (FAPESP) 2021/08134-2 (Scheffer, FR); Research Assistant level III by the São Paulo Research Foundation (FAPESP) 2020/14927-2 (Maekawa, AS); Productivity Grant by The Brazilian National Council for Scientific and Technological Development (CNPq) 306970/2018-0 (Velho, PENF); Postdoctoral Scholarship by The São Paulo Research Foundation (FAPESP) 2018/12565-6 (Drummond, MR).

## Conflicts of interest

The authors declare no conflicts of interest.
